# Characteristics and outcomes of patients hospitalized with COVID-19 at a tertiary hospital in Nigeria

**DOI:** 10.4314/ahs.v23i1.9

**Published:** 2023-03

**Authors:** Olukemi Adekanmbi, Olayinka Ilesanmi, Olusola Idowu, Arinola Esan, Yemi R Raji, Adeola Fowotade, Olubunmi Ogunlade, Adegboyega Akere, Oluwaseun Ololade, Kehinde Ojifinni, Olurotimi Akinola, Akintunde Orunmuyi, Uwom Eze, Victor Akinmoladun, Abiodun Adeoye, Akindele Adebiyi, E Oluwabunmi Olapade-Olaopa, Jesse A Otegbayo, Kayode Osungbade

**Affiliations:** 1 Department of Medicine, College of Medicine, University of Ibadan, Ibadan, Nigeria; 2 Department of Medicine, University College Hospital, Ibadan, Nigeria; 3 Department of Community Medicine, College of Medicine, University of Ibadan, Ibadan, Nigeria; 4 Department of Community Medicine, University College Hospital, Ibadan, Nigeria; 5 Department of Anaesthesia, College of Medicine, University of Ibadan, Ibadan, Nigeria; 6 Department of Anaesthesia, University College Hospital, Ibadan, Nigeria; 7 Department of Medical Microbiology, College of Medicine, University of Ibadan, Ibadan, Nigeria; 8 Department of Emergency Medicine, University College Hospital, Ibadan, Nigeria; 9 Department of Nuclear Medicine, College of Medicine, University of Ibadan, Ibadan, Nigeria; 10 Department of Pathology, University College Hospital, Ibadan, Nigeria; 12 Department of Oral and Maxillofacial Surgery, College of Medicine, University of Ibadan; 13 Department of Oral and Maxillofacial Surgery, University College Hospital, Ibadan; 14 Institute of Cardiovascular Disease, College of Medicine, University of Ibadan, Ibadan, Nigeria; 15 Department of Surgery, College of Medicine, University of Ibadan, Ibadan, Nigeria; 16 Department of Surgery, University College Hospital, Ibadan, Nigeria; 17 Department of Health Policy and Management, College of Medicine, University of Ibadan, Ibadan; 18 Disease Surveillance Unit, University College Hospital, Ibadan, Nigeria

**Keywords:** COVID-19, COVID-19 mortality, SARS CoV-2

## Abstract

**Background:**

Data regarding the features and outcomes of hospitalized COVID-19 patients in Africa are increasingly available.

**Objectives:**

To describe socio-demographic, clinical and laboratory characteristics and outcomes of COVID-19 patients.

**Methods:**

A cross-sectional study of 86 adult patients hospitalized with COVID-19 between March and November 2020. Characteristics were described in survivors and non-survivors.

**Results:**

Mean age was 60.9±16.1 years, 53(61.6%) were male. Co-morbidities were found in 77(89.5%) patients. On severity, 6(7%) were mild, 23(26.7%) moderate, 51(59.3%) severe and 6(7%) critical. Oxygen saturation and respiratory rate were 71±22% and 38±11/minute in non-survivors and 90±7% and 31±7/minute in survivors respectively (p<0.001, p<0.001)). Overall mortality was 47.7% with no death among patients with mild disease and deaths in all patients with critical disease. Duration of hospitalization was 2.0(1.0-4.5) days in those who died and 12(7.0-15.0) days in those who survived (p<0.001). Of the 42 patients that received dexamethasone, 11(26.2%) died, while 31(73.8%) survived (p=<0.001).

**Conclusion:**

Most of the patients had co-morbidities and there was high mortality in patients with severe and critical COVID-19. Mean oxygen saturation was low and respiratory rate high overall. Factors associated with mortality included: Significantly greater hypoxia and tachypnea, less dexamethasone use and shorter hospitalization.

## Introduction

Africa appears to have been the final frontier in the spread of COVID-19 as the disease seemed to have arrived on the African continent a while after it spread through other parts of the world 1, 2. Fortunately, though unexpectedly, reported mortality in Africa appears lower than in other regions 3. The reasons for this are not clear and several postulations have been put forth 4–6.

The first known case of SARS CoV-2 infection in Nigeria was detected on 27^th^ February 2020 in an Italian national^7^. In January 2022, almost 2 years after the first case was detected in Nigeria, over 3,800,000 tests had been performed, 246,195 cases identified, and 3,066 deaths recorded.^8^ At the same time, 298,915,721 cases and 5,469,303 deaths have been recorded globally; while 9,756,457 cases and 228,856 deaths were recorded in Africa 9, 10. The true number of cases in Africa is believed to be much higher and there are data to support this 11.

Nigeria was one of the first countries in sub-Saharan Africa to report a case of COVID-19 and had about 5.46% of cumulative cases in the WHO African Region as of March 2021 12. Nigeria accounts for almost 20% of the continental population 13.

While data are available on characteristics and outcomes of patients with COVID from other parts of the globe, the data on COVID-19 from sub-Saharan Africa is sparse, especially for severely ill patients. We describe the socio-demographic, clinical and laboratory characteristics as well as outcomes of patients hospitalized at a tertiary hospital during the first wave of the COVID-19 pandemic in Nigeria.

## Methods

### Study design

This was a cross-sectional study of 86 patients diagnosed with COVID-19 and admitted at the University College Hospital (UCH) in Ibadan, South-west Nigeria between 27^th^ March 2020 and 3^rd^ November 2020. The follow up continued until discharge of the last of these patients on 17^th^ November 2020.

### Study setting

Ibadan in Oyo State, is located 140 km from Lagos, Nigeria's COVID epicentre.

### Study site

The University College Hospital is the oldest tertiary hospital in Nigeria and is an 800-bed facility which, at the start of the pandemic, had a 4-bed Infectious Disease treatment center.

### Eligibility criteria

All patients at the UCH Infectious Disease (ID) Center were eligible to be enrolled in the study. Admission into the UCH ID center was restricted to those who were confirmed by polymerase chain reaction (PCR) to be infected with SARS-CoV-2 and required admission for acute care. Patients who were admitted to other wards in the hospital with other diagnoses and were subsequently diagnosed with COVID-19 during that admission were included. Seven patients whose in-patient records were incomplete or could not be located at time of data collection were excluded from the study.

### Investigations

Baseline investigations included a full blood count (FBC), serum electrolytes, urea and creatinine (E/U/Cr) and blood glucose levels. DDimer, C reactive protein (CRP), liver function tests (LFT), hemoglobin A1C, (HbA1C) were also performed as indicated. Chest radiography and computed tomography (CT) of the chest were performed where possible. Cultures of blood sputum and urine, Xpert MTB Rif, blood film for malaria parasite and other investigations were performed as indicated. While some of these investigations were paid for by the state government, payment for others were out-of-pocket and this, sometimes, precluded getting these tests done.

### Treatment guidelines

Treatment was initially guided by an institutional protocol based on guidelines developed by the Nigerian Center for Disease Control (NCDC) for COVID-19 case management and the World Health Organization (WHO) protocol for management of Severe Acute Respiratory Illness (SARI) 14, 15. The institutional protocol evolved over the time covered in the study to reflect newly available evidence and changes in NCDC and WHO guidelines. At the outset, all patients received Vitamin C and zinc. Vitamin D was added to the protocol a bit further into pandemic as were low molecular weight heparin, proton pump inhibitors and dexamethasone (for those with oxygen saturation less than 94%). The UCH protocol included the use of Chloroquine or Hydroxychloroquine at the beginning of the pandemic if there were no contra-indications. Antibiotics (azithromycin +/- ceftriaxone) were also used based on the clinician's assessment of disease severity and suspicion for or evidence of secondary bacterial infection.

### Data collection and analysis

Socio-demographic, clinical and laboratory parameters as well as length of stay and mortality were collected from routine paper-based records. Comorbidities identified in the study were hypertension (history of previously diagnosed hypertension or current blood pressure >140/90mmHg; diabetes mellitus (history of previously diagnosed diabetes, fasting plasma glucose of >126mg/dL or Hemoglobin A1C>6.5%, heart disease (previous diagnosis of any heart pathology); kidney disease (previous diagnosis of any kidney disease, elevated serum creatinine or reduced estimated glomerular filtration rate) and cancer. Frequency, and proportion of each of these were presented. Patients having at least one of the comorbidities were categorized as having comorbidity in the overall classification. The patients were classified as mild, moderate, severe, and critical according to the WHO criteria for disease severity 15. Using these criteria, patients with severe disease in our study had respiratory rate>30 breaths per minute or SpO^2^<93% in room air while those with critical disease had acute respiratory distress syndrome (ARDS). The initial vital signs and laboratory data obtained during admission were abstracted and analysed. Clinical and other variables were compared between those who died during hospital admission and those who remained alive. Outcomes of interest were length of hospital stay and in-hospital mortality.

Data was entered into and analysed with SPSS version 25^16^. Age and other parametric variables were summarized using mean, while frequencies and percentages were used for categorical variables. Mean age and other parametric variables (vital signs) were compared between dead and alive patients using the independent t test. The median of laboratory result parameters was compared using Mann-Whitney U test due to its skewness. Chi-square test was used to assess differences between socio-demographic characteristics, baseline co-morbidities, symptoms, the type of treatment and outcome status (dead or alive). P-values <0.05 were statistically significant.

The study was approved by the Institutional Review Board of the University College Hospital and the University of Ibadan (UI/UCH ethics committee assigned number UI/EC/20/410). Patient informed consent was waived given the retrospective use of de-identified data.

## Results

The mean age of all 86 patients in this study was 60.9±16.1 years and 53 (61.6%) of the patients were males. Minimum age was 20 years and the maximum 86 years. The 60–69-year age group accounted for 29 (33.7%) of all the patients. Also, 51 (59.3%) patients had severe COVID-19 at presentation while 6 (7%) each classified as having mild and critical COVID-19 disease (Table 1). Among the patients, 83 (96.5%) did not report a known prior exposure to a patient with COVID-19. Seventy-seven (89.5%) patients had at least one co-morbidity with 54 (64.3%) having hypertension and 36 (42.9%) having diabetes mellitus. Among COVID-19 patients with heart disease, 4 (23.5%) died while 13 (76.5%) lived (χ 2 = 4.952, p = 0.026). Only one patient had HIV in our study and another patient had pulmonary tuberculosis co-infection diagnosed and treated during admission.

**Table 1:** Socio-demographic and baseline characteristics of patients hospitalized for COVID-19 at the University College Hospital, Ibadan

Characteristics	Overall total n (%)	Dead n (%)	Alive n (%)	Chi-square	p-value
**Age mean (SD)** **(Years)**	60.9±16.1	61.8±17.4	60.0±14.9	0.532*	0.596
**Age group**					
20–29	4 (4.7)	2 (50.0)	2 (50.0)	**	0.26
30–39	5 (5.8)	4 (80.0)	1 (20.0)		
40–49	11 (12.8)	3 (37.5)	8 (62.5)		
50–59	12 (14.0)	4 (33.3)	8 (66.7)		
60–69	29 (33.7)	13 (44.8)	16 (65.2)		
70–79	11 (12.8)	8 (72.7)	3 (27.3)		
80+	14 (16.3)	7 (50.0)	7 (50.0)		
**Sex**					
Male	53 (61.6)	24 (35.3)	29 (64.7)	0.317	0.574
Female	33 (38.4)	17 (51.5)	16 (48.5)		
**Contact with** **Known Positive**					
Yes	3 (3.5)	0 (0.0)	3 (100.0)	2.832	0.092
No	83 (96.5)	41 (49.4)	42 (50.6)		
**Comorbidities**					
Presence of any Comorbidity	77(89.5)	41(53.2)	36(46.8)	0.250	0.617
Hypertensive (n=84)	54 (64.3)	24 (44.4)	30 (55.6)	0.239	0.625
Diabetic (n=84)	36 (42.9)	20 (55.6)	16 (44.4)	2.110	0.146
Heart disease	17 (19.8)	4 (23.5)	13 (76.5)	4.952	**0.026**
Kidney disease	17 (19.8)	7 (41.2)	10 (58.8)	0.359	0.549
Cancer	7 (8.1)	4 (57.1)	3 (42.9)	0.274	0.601
**Severity of** **COVID-19**					
Mild	6 (7.0)	0(0.0)	6(100.0)	**	0.079
Moderate	23(26.7)	6(26.1)	17(73.9)		
Severe	51(59.3)	29(56.9)	22(43.1)		
Critical	6(7.0)	6(100.0)	0(0.0)		

* Independent t-test

Overall, 41(47.7%) patients died during hospitalization. Twenty-nine (56.9%) patients with severe disease died compared to 6 (26.1%) with moderate disease, and 6 (100%) of the critically ill patients (χ^2^=18.075, p = <0.001)

Also, 10 (100%) of persons who had spent less than 24 hours on admission died, compared to 6 (15.0%) who had spent more than 7 days on admission (χ^2^=39.968, p = <0.001).

Other socio-demographic baseline characteristics and outcomes of the hospitalized patients are shown in Table 1.

The main reported symptoms were shortness of breath in 60 (69.8%) patients, cough in 57(66.3%) patients, and fever in 53(61.6%) patients. Significantly more patients with shortness of breath survived than died (34 (56.7%) versus 26 (43.3%)) (χ^2^ = 6.433, p = 0.011). Also, 52 (60.5%) patients presented with fatigue and 34 (65.4%) of them died, while 18 (34.6%) lived (χ^2^ = 8.992, p = 0.003) (Table 2).

**Table 2:** Frequency of symptoms among COVID-19 positive patients by mortality among hospitalized COVID-19 patients at the University College Hospital, Ibadan

Symptoms at the time of admission	Overall	Dead	Alive	Chi-square	p-value
	n (%)	n (%)	n (%)		
Shortness of breath	60 (69.8)	26(43.3)	34(56.7)	6.433	**0.011**
Cough	57 (66.3)	30(52.6)	27 (47.4)	0.006	0.937
Fever	53(61.6)	25(47.2)	28(52.8)	1.472	0.225
Fatigue	52 (60.5)	34(65.4)	18(34.6)	8.992	**0.003**
Sputum production	26 (30.2)	18(69.2)	8(30.8)	4.269	**0.039**
Altered Sensorium	16 (18.6)	5(31.3)	1(68.8)	3.50	0.061
Muscle Aches and Pain	11 (18.6)	8(72.7)	3(27.3)	2.105	0.147
Diarrhea	10 (11.6)	6(60.0)	4(40.0)	0.267	0.605
Chest Pain	8 (9.3)	4(50.0)	4(50.0)	0.019	0.890
Sore throat	7 (8.1)	5(71.4)	2(28.6)	1.115	0.291
Abdominal Pain	7 (8.1)	5(71.4)	2(28.6)	1.115	0.291
Nausea or Vomiting	7 (8.1)	5(71.4)	2(28.6)	1.115	0.291
Headache	7 (8.1)	4(57.1)	3(42.9)	0.071	0.790
Loss of Taste	7 (8.1)	5(71.4)	2(28.6)	1.115	0.291
Loss of Smell	4 (4.7)	3(75.0)	1(25.0)	0.865	0.352
Nasal Congestion	1 (1.2)	0(0)	1(100.0)	1.110	0.292

Table 3 shows the vital signs on admission, laboratory investigations and treatment interventions of COVID-19 patients. The mean oxygen saturation at presentation among those who died was significantly lower, 71±22% compared to 90±7% among those who lived (p<0.001). The mean respiratory rate per minute of patients who died was significantly higher (38±11) than that of those who lived (31±7) (p = <0.001).

**Table 3:** Vital signs on admission, laboratory investigations and therapeutic interventions of patients hospitalized with COVID-19 at the University College Hospital, Ibadan

Vital signs on admission	Overall Mean ± SD	Mean ± SD Dead	Mean ± SD Alive	p-value
Pulse rate (beats/min) (n=85)	101±18	102±22	100±15	0.480
Systolic BP (mmHg) (N=85)	135±35	137±44	135±25	0.42
Diastolic BP (mmHg) (N=84)	80±20	81±23	79±14	0.518
Oxygen saturation (%) (N=85)	81±19	71±22	90±7.0	**<0.001**
Respiratory rate (breaths/min) (N=86)	35±10	38±11	31±7	**<0.001**
Temp (°c) (n=85)	37.0±0.2	37.0±1.0	37.1±1.1	0.787
GCS (n=85)	14±4	12±4	15±4	**<0.001**
Duration between the first symptom and presentation (days)	9±6	7±5	10±6	**0.017**
**Laboratory** **Investigations**	**Median (IQR)**	**Dead** **Median (IQR)**	**Alive** **Median (IQR)**	**p-value**
WBC (cells/µl) (n=67)	10.2 (7.4 -13.8)	12.1 (7.4-17.8)	9.8 (7.5–12.7)	0.322
ANC (cells/µ/l) (n=66)	7.7 (4.8-10.9)	9.3 (5.2–13.3)	7.7 (4.8–10.0)	0.658
ALC (cells/µl) (n=65)	1.5 (0.8-2.0)	1.6 (0.9–2.7)	1.4 (0.7–2.0)	0.995
HGB (g/dl) (n=69)	11.5 (10.0–13.3)	11.0 (7.35–13.0)	11.9 (10.1–13.6)	0.135
PLATELET (cells/ µl) (n=65)	236,500 (157,750–312,750)	228,000 (113,000–352,000)	239,000 (181,000–310,000)	0.595
Creatinine (mg/dl) (n=60)	1.1 (0.8–1.6)	1.21 (0.98–3.15)	1.1 (0.78–1.50)	0.202
DDimer (µg/ml) (n=29)	2.75 (0.93–8.0)	4.3 (1.7–5.2)	1.1 (0.8–9.6)	1.000
CRP mg/L(n=17)	62 (16.0–166.2)	233.9 (105.7–233.9)	35.6 (10.4–148.4)	0.176
Fasting Blood Sugar (mg/dl) (n=12)	129.5 (100.0–222.0)	210.0 (144.0–252.5)	120.0 (96.0–138.0)	0.268
Random Blood Sugar (mg/dl) (n=39)	194.0 (127.0–268.0)	201.0 (124.0–268.0)	186.0 (138.0–253.3)	0.855
Duration of hospitalization n=86	6.0 (2.0–13.0)	2.0 (1.0–4.5)	12.0 (7.0–15.0)	**<0.001**

WBC- White Blood Cell Count, ANC- Absolute Neutrophil Count, ALC- Absolute Lymphocyte Count, CRP – C-Reactive Protein, HGB- Haemoglobin

The overall median white blood cell (WBC) count (SD) among our study population was 10.2 cells/µl (7.4-13.8 cells/µl). It was higher (though not significantly), 12.1 cells/ µl (7.42-17.8 cells/ µl) in patients that died than in those who did not (9.8 cells/ µl [7.5-12.7 cells/ µl]) (p=0.728). Median absolute lymphocyte count (SD) was 1.5 cells/ µl overall, 1.6 (0.9-2.0) cells/ µl in those who died and 1.4 (0.7-2.0) cells/ µl in those who survived (p=0.995).

Only 28 (32.6%) patients had a chest radiograph, 19(67.9%) had patchy infiltrates, 9(47.4%) of those with this finding died compared to 1(11.1%) of those without (p=0.061). In addition, 1 each had lung abscess, pleural effusion, cardiomegaly, and a normal chest radiograph. Five had CT scan of the chest which showed ground glass opacities in 3 patients and consolidation in the other 2. Forty-two (48.8%) of the patients in the study received dexamethasone and while 11(26.2%) of these patients died, 31(73.8%) survived (χ2=15.189, p<0.001) (Table 4).

**Table 4:** Treatment received by patients hospitalized with COVID-19 at the University College Hospital, Ibadan

Treatments	Overall n (%)	Dead	Alive	Chi square	P value
Chloroquine and/or hydroxychloroquine	8(9.3)	4 (50.0)	4 (50.0)	0.019	0.890
Dexamethasone	42(48.8)	11 (26.2)	31 (73.8)	15.189	**<0.001**
Enoxaparin	64(74.4)	26 (40.6)	38 (59.3)	4.984	**0.026**
Angiotensin Converting Enzyme Inhibitor	20(23.3)	5 (25.0)	15 (75.0)	5.371	**0.020**
Zinc	54(62.8)	17 (31.5)	37 (68.5)	15.254	**<0.001**
Vitamin C	57(66.3)	19 (33.3)	31(73.8)	15.189	**<0.001**
Vitamin D	51(59.3%)	16(31.4)	35(68.6)	13.350	**<0.001**
Ceftriaxone	63(73.3%)	28(44.5)	35(55.6)	0.985	0.321
Azithromycin	57(66.3)	21(36.8)	36(63.2)	7.951	**0.005**

Overall, 57(66.3%) received vitamin C of which 19(33.3%) died and 38(66.7%) did not (χ2=13.936, p<0.001). Overall, 74 (86%) required oxygen, 53(61.6%) received oxygen via one of either nasal prongs or non-breather mask, while 21(24.4%) had both, each at some point during hospitalization. One patient had oxygen via continuous positive airway pressure (CPAP) and survived while two were intubated and mechanically ventilated (and died).

**Figure 1:**
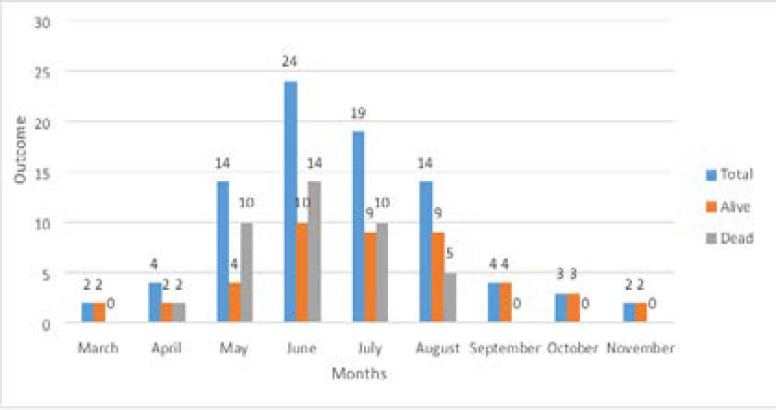
Mortality by month in 2020 during the first wave of COVID-19 among hospitalized COVID19 patients at the University College Hospital, Ibadan

Figure 1 shows mortality among patients in our study by month of admission. The highest number of COVID-19 cases were admitted in June 2020 with a total of 24 COVID-19 admissions, of which 14 died and 10 survived. The month of November 2020 had the lowest number of COVID-19 admissions with 2 persons admitted, both of whom survived.

## Discussion

This study provides a description of more severely ill COVID-19 patients by survival status than have been previously reported from Nigeria. With a mean age of 60.9 years and more than 60% of the patients aged above 60 years, this is an older group of patients than were reported elsewhere in Africa with a median age of 34.2 years in Uganda and mean ages of 38.1, 46.2 and 43 years in Nigeria^17^,^18^,^19^,^20^. Disease severity among patients in our study population weighed more towards severely ill patients. Our study mirrors more closely the age and more severe disease reported for hospitalized patients in China, Europe and North America and provides some much-needed information about the more severe end of the COVID-19 spectrum in this part of the world 21–23. These are the patients that can overwhelm the already strained health systems in resource-limited settings.

Expectedly for the age distribution of our study population, almost 90% of our patients had at least one co-morbidity with hypertension and diabetes being the most frequently reported as has been reported elsewhere. This is higher than reported by Otuonye and Osibogun in Nigeria who reported 49.4% and 22.5% prevalences of co-morbidities respectively 19, 24. As found in this study, a New York, USA study reported 56.6% and 28.8% 25, while another from Detroit, USA reported 63.7% and 38.4% prevalences for hypertension and diabetes respectively^22^. Kirenga from Uganda,^17^ however reported a lower prevalence (10.7% each) for hypertension and diabetes while Nachega in the Democratic Republic of Congo 1, reported 25.4% prevalence for hypertension. Similarly, to these, Erinoso in Nigeria reported 16.8% hypertension and 5.2% diabetes 26. This underscores the dissimilarity between previously studied COVID patients in Africa and ours. In our study, a significantly higher proportion of patients with heart disease survived than died. The reason for this is not clear and is an unexpected finding. Presence of other co-morbidities do not differ significantly between those who died and those who survived in our study, this may be due to our small sample size.

The most frequently reported symptoms in our study were shortness of breath, cough, fever, and fatigue and this is in keeping with reports from a meta-analysis involving 3600 patients from 43 studies in China as well studies conducted in Nigeria 19, 20,26, 27. It is not surprising that given extremely limited capacity for admission during the first wave of the pandemic, the need for oxygen therapy (which is logistically difficult to deliver in the home setting locally) was a leading indication for admission and hence shortness of breath was the commonest symptom among patients who were hospitalized. Shortness of breath was significantly less common among patients who died. Shortness of breath is however subjective and may not reflect a true need for oxygen therapy as does oxygen saturation. Fever on admission was detected in 61.6% of our patients. Fever in hospitalized COVID-19 patients has been reported anywhere from 21.4% as reported by Kirenga et al in Uganda to 98.6% by Guan et al in China 3, 17, 28, 29. This wide variation in the prevalence of fever at presentation might be due to the possible use of antipyretic medication prior to presentation.

Apart from respiratory symptoms and fever, there was a wide range of symptoms recorded in this study. Fatigue was also quite common and occurred significantly more frequently in non-survivors than survivors. Neurological features such as headache, anosmia and ageusia as well as gastro-intestinal symptoms were relatively rare. Headache and gastrointestinal symptoms were also relatively uncommon in early studies from China but Barry et al in Saudi Arabia reported 37.5% gastrointestinal symptoms in their patients 3, 28, 30.

With a mean oxygen saturation of 90±7% in those who survived and 71±22% in those who died (p<0.001), degree of hypoxia was significantly worse in those who died in our study. Ayinbuomwan in Nigeria had similar findings where more patients who had normal oxygen saturation at presentation survived 31. Majority of our hospitalized patients required oxygen therapy of some sort and provision of oxygen should be a key consideration for policy makers in terms of preparedness for acute care of the hospitalized COVID-19 patient. If validated in larger studies actually designed to assess for risk factors for mortality, higher respiratory rates, 38±11 in those that died than those who survived (31±7) could point to its use as a readily accessible predictor of disease severity where pulse oximetry may not be readily available.

Higher total WBC counts, CRP, DDimer, and blood glucose levels were more frequently recorded in patients that died than in the survivors. These differences between survivors and non-survivors were not statistically significant probably because these data were available for only a limited number of patients but have all been widely reported as markers of severe disease 32–34. As has been well reported for COVID-19, we found overall median lymphocyte count to be low at 1.5 cells/µl even though in our study, survivors had lower lymphocyte counts than those who died during admission (though not statistically significant) which is contrary to earlier reports of lymphopenia being associated with severe disease 35, 36. Lower platelet counts and hemoglobin levels were seen in those who died, this observation of thrombocytopenia in COVID-19 has also been previously reported 37.

Dexamethasone, zinc, azithromycin and low molecular weight heparin were all used significantly more frequently in the patients that survived than those that did not. Dexamethasone was introduced (for patients with oxygen saturation less than 94%) into our treatment protocol after the findings of the RECOVERY trial became available in July 2020 38. This was followed by a reduction in mortality among our patients and our study is one of the first in sub-Saharan Africa to report this observation. Most other available published studies from sub-Saharan Africa at the time of writing predate the inclusion of dexamethasone in treatment protocols. The RECOVERY trial demonstrated that dexamethasone has mortality benefit at 28 days in COVID-19 patients requiring oxygen supplementation. Anticoagulant therapy is believed to be beneficial for treating the thromboembolic phenomena associated with COVID-19 and was less frequently used among patients that died in our study 37.

Overall mortality in our study was 47.7%. This is quite high and agrees with what has been reported from different regions in comparable patient populations. Nachega in the Democratic republic of Congo and Parker in South Africa reported around 50% mortality among severely and critically ill COVID-19 patients 39, 40. A sizeable proportion of our patients (66.3%) were severely or critically ill and would have met criteria for intensive care unit (ICU) admission if the facilities were readily available. A large study from Nigeria reported 4.3% mortality in a relatively younger cohort than ours 20. Studies from Europe and the US reported overall mortality between 25 and 30% for varying degrees of disease severity. Late presentation also likely plays a key role in mortality as many who died within 24 hours of presentation were critically ill at the time of presentation.

Mortality is not significantly different across the different age groups and gender in our study and is likely due to the modest sample size. Studies elsewhere have reported higher mortality in older patients^11^.

## Limitations

Our study has some limitations. It is a descriptive study and as such is not sufficient to identify predictors of mortality but rather a comparison of variables in survivors and non-survivors. The study was conducted at a single center which would limit the generalizability of the findings. We cannot also rule out the possibility of referral filter bias being a tertiary hospital. The relatively small sample size also might have led to the underestimation of certain expected differences. In addition, the laboratory data were incomplete because in the earlier part of the pandemic, biochemical and other laboratory testing could not be readily performed in the hospital's laboratories because of biosafety concerns. The use of only paper records and non-standardized method of documentation in medical records might have affected the quality of data available for extraction. Our study only took into consideration COVID-19 patients who were hospitalized. Therefore, findings from this study cannot be generalized to patients who do not require hospitalization for management of COVID-19.

## Conclusion

In this cross-sectional study of hospitalized COVID-19 patients in Nigeria, there was a high prevalence of comorbidity and severe or critical disease. Respiratory symptoms with requirement for oxygen therapy was a frequent need. Hypoxia and tachypnea occurred more frequently in patients that died and following validation may serve as early pointer for healthcare workers to those at risk for work outcomes. Dexamethasone in patients with need for oxygen therapy was more frequently used in survivors than non survivors. Hospitalizations lasting less than 24 hours all resulted in death which may suggest late presentation and/or referral. Given that mortality was high in patients with severe and critical disease, resources to care for such patients are essential for better outcomes. Larger studies designed to explore factors predictive of mortality such as age, co-morbidity and therapeutic interventions are needed in this setting.
